# A failed RCT to determine if antibiotics prevent mastitis: Cracked nipples colonized with *Staphylococcus aureus*: A randomized treatment trial [ISRCTN65289389]

**DOI:** 10.1186/1471-2393-4-19

**Published:** 2004-09-16

**Authors:** Lisa Helen Amir, Judith Lumley, Suzanne M Garland

**Affiliations:** 1 Mother and Child Health Research, La Trobe University, Melbourne, Australia; 2 Department of Microbiological Research, Microbiology and Infectious Diseases, Royal Women's and Children's Hospitals, Melbourne, Australia

## Abstract

**Background:**

A small, non-blinded, RCT (randomised controlled trial) had reported that oral antibiotics reduced the incidence of mastitis in lactating women with *Staphylococcus aureus *(*S. aureus*)- colonized cracked nipples. We aimed to replicate the study with a more rigorous design and adequate sample size.

**Methods:**

Our intention was to conduct a double-blind placebo-controlled trial to determine if an antibiotic (flucloxacillin) could prevent mastitis in lactating women with *S. aureus*-colonized cracked nipples. We planned to recruit two groups of 133 women with *S. aureus-*colonized cracked nipples.

**Results:**

We spent over twelve months submitting applications to five hospital ethics committees and seven funding bodies, before commencing the trial. Recruitment to the trial was very slow and only ten women were randomized to the trial after twelve months, and therefore the trial was stopped early.

**Conclusions:**

In retrospect we should have conducted a feasibility study, which would have revealed the low number of women in these Melbourne hospitals (maternity wards and breastfeeding clinics) with damaged nipples. The appropriate use of antibiotics for breastfeeding women with cracked nipples still needs to be tested.

## Background

Mastitis is a common problem for breastfeeding women [1,2]. Before planning a trial to reduce the number of lactating women who develop mastitis, we reviewed the literature to identify factors that may be associated with mastitis and to examine previous trials. A relatively small number of trials was identified which included mastitis as one of the outcome measures (see Table) [3-13]. Using historical controls, prophylactic topical penicillin ointment was found to be ineffective [3], while hand disinfectant at the mother's bedside appeared to reduce mastitis [7]. A Finnish study examined "breast massage" (which appears to be a variation of "nipple toughening") and found no impact of this practice on mastitis [10].

**Table 1:** Trials to prevent mastitis

**Author, date, country**	**Subjects**	**Aim**	**Control(presence/absence/type)**	**Intervention**	**Sample size**	**Outcome: mastitis**
Hesseltine et al 1948, USA[3]	Patients at the Chicago Lying-In Hospital: July – Sept 1946	Does topical penicillin ointment on mother's nipples prevent mastitis?	Historical controls: July 1933 to Dec 1946	Penicillin ointment (2,000 units per treatment) on nipples after feeds (6–8 weeks)	Intervention 865; Controls 40,629	Intervention: 53 women with mastitis, 6.1%, and 18 with abscess, 2%; Control: 210 women with abscess, 0.51%
Sasse 1973, Germany (in German)[4]	Postnatal women in the Frauenclinik der Freien Universitat Berlin-Charlottenburg, 1967	Does an antibiotic spray to mother's nipples prevent mastitis?	Historical controls	Nabectin Puder Spray(neomycin and bacitracin) applied to nipples, plus hand disinfection for nurses and mothers before handling breasts.	Intervention130; Controls100	Intervention: 7% mastitis by 2 months; Control: 23%
Berger & Pusteria 1981 Switzerland[5]	Postnatal women in the Women's Hospital, University of Berne(reported in 1962 [22])	Does nipple ointment prevent mastitis?	One group used a nipple ointment without the active ingredient. Not a RCT.	Six nipple ointments:		
				(a) boric-acid Vaseline with Peruvian balsam,	(a) 1,000	(a) 1.5%
				(b) chlortetracycline,	(b) 1,000	(b) 0.7%
				(c) chlorquinadol ointment,	(c) 1,000	(c) 0.4%
				(d) base of chlorquinadol ointment (without active ingredient),	(d) 1,000	(d) 0.4%
				(e) calcium pantothenicum,	(e) 2,000	(e) 0.8%
				(f) dihydrofolliculin benzoate and tyrothrycin	(f) 1,500	(f) 0.5%
Kovalev 1990, Russia (in Russian)[6]		Does treating cracked nipples with laser therapy prevent mastitis?	Unclear from abstract	Laser treatment to damaged nipples	329 women with damaged nipples	Intervention reduced mastitis from 18.6% to 3.7%
Sytnik 1990, Russia [8] (in Russian)[8]		Does bifidobacterium prevent mastitis?	Unclear from abstract	Bifidobacterium	160 women	Mastitis reduced from 6.88% to 1.25%
Peters and Flick-Fillies1991, Germany[7,23]	Postnatal women in St Hildegardis Hospital, Mainz, 1989–1991	Does the use of bedside hand disinfectant prevent mastitis?	Historical controls: 12 months (Sep 1989-Jun 1990, May-Jun 1991)	Bed-side disinfectant dispensers: 12 months (Jul 1990-April 1991, Jul-Aug 1991)	Intervention: 1095; Control 1230	Intervention 8 women, 0.65%; Control 32 women 2.9%; p <0.001
Waldenstrom and Nilsson 1994, Sweden[9]	Women giving birth at South Hospital, Stockhom	Is birth centre care beneficial for breastfeeding? Does it increase duration and reduce complications (including mastitis)?	RCT	Birth centre care compared to standard care	Intervention 617; Control 613.	Postal questionnaire 2 months postpartum. "Milk stasis" (fever and swelling, redness and tenderness in one of the breasts): Intervention 26%; Control 19% (p = 0.002). "Mastitis" (infective breast treated with antibiotics): Intervention 1%, Control < 1% (p = 0.07)
Jonsson & Pulkkinen 1994, Finland[10]	Women in South-West Finland	Does antenatal / postnatal breast massage prevent mastitis?	Concurrent controls.	"Breast massage with the hands, a brush, a coarse towel or a sponge before and / or after delivery"	Intervention 255, Control 400.	Questionnaire 5–12 weeks postpartum at outpatient visit. Overall incidence of mastitis was 24%. No difference in incidence of mastitis (no details given). "This physical training of the nipples neither decreases or increases the frequency of mastitis" (p86)
Evans et al 1995, Australia[11]	Postnatal women at Flinders Medical Centre, Adelaide	Does prolonged feeding on one breast per feed reduce breastfeeding complications, including mastitis?	Historical controls: 5 months	Advice to feed from one breast per feed and only offer the second breast if the baby still showed signs of hunger rather than standard care of both breasts at each feed: 5 months	Intervention 150; Control 152	Telephone interview at 6 months postpartum: Intervention 15%; Control 18%
Gunn et al 1998, Australia[12]	Women giving birth in one metropolitan hospital and one rural hospital in Victoria, 1995	Does an early visit to a general practitioner reduce problems (including mastitis) compared to the standard six-week postnatal visit?	RCT	General practitioner visit at one week compared to standard six week visit	Intervention 232; Control 243	Postal questionnaire at 3 months. Intervention 11.6%; Control 15.6% (Odds Ratio 0.71, 95%CI: 0.42, 1.20)
Livingstone & Stringer, Canada[13]	Women attending the Vancouver Breastfeeding Center with a cracked nipple and *S. aureus *positive culture.	Are oral or topical antibiotics more effective in the treatment of *S. aureus*-colonized cracked nipples than standard care?	RCT (not blind to treatment group or outcome)	4 groups:		Assessment at 7 days. Oral antibiotics: 1/19, 5%; Other groups: 16/65 (25%) (Fisher exact 0.1)
				(a) oral antibiotics	(a) 19	
				(b) topical mupirocin	(b) 25	
				(c) topical fusidic acid	(c) 17	
				(d) standard care	(d) 23	

The authors of one trial were convinced that their intervention was effective, despite methodological difficulties [13,14]. Livingstone and Stringer conducted a randomised trial for women with cracked nipples with positive cultures for *Staphylococcus aureus *(*S. aureus)*, in Canada[13]. They compared topical antibiotics, oral antibiotics and "optimal breastfeeding advice" and found better improvement in nipple healing in the women given oral antibiotics. In addition, they found 16 women out of 65 (25%) given non-systemic treatment developed mastitis within 7 days, compared to 1 of 19 women (5%) given systemic antibiotics (chi-square, p = 0.065) [not 0.005 as stated in their abstract]. The authors have concluded that cracked nipples colonized with *S. aureus *should be "treated aggressively with systemic antibiotics". However, the chi-square test used by the authors is inappropriate because one cell contains an expected value less than 5. Using Fisher's exact test, the p value is 0.10 [15].

As the Livingstone and Stringer trial had been published in a major lactation journal and was likely to be very influential in practice [13], it needs to be replicated in a more rigorous manner in order to assess the usefulness and safety of the intervention. Our intention was to replicate that study with an adequate sample size, rigorous definitions of nipple damage and mastitis, and double blinding of the intervention.

## Methods

The aim of our study was to prevent mastitis in breastfeeding women with cracked nipples colonized with *S. aureus*. A randomised controlled trial was conducted: participating women were randomized to receive a seven day course of either an oral antibiotic (flucloxacillin) or identical placebo capsules. A follow-up visit was arranged one week after recruitment for women with positive nipple culture for *S. aureus*. Women with negative nipple culture were followed up by telephone at one week. All women received a final telephone interview at six weeks.

The primary outcome was the incidence of mastitis in each group in the week following recruitment. In the study by Livingstone and Stringer [13] 30% of women with *S. aureus*-colonized cracked nipples who received only breastfeeding advice developed mastitis within one week. In order to detect a 50% decrease in incidence, ie mastitis occurring in 15% of women receiving oral antibiotics, a sample size of 133 women in each group is required, with 95% confidence and 80% power. Sample size was calculated using Epi-Info 6.

A previous study in Australia found that 62% (13/21) of cultures from breastfeeding women with cracked nipples were positive for *S. aureus *[16]. An earlier study by Livingstone and colleagues found that 54% of cracked nipples of mothers with infants younger than one month were positive for *S. aureus *(27/50) [17]. Assuming that 50% of cracked nipples are positive for *S. aureus*, we would need 133 × 2 × 2 = 532 women with cracked nipples, to recruit two groups of 133 women with *S. aureus*-colonized cracked nipples. To allow for loss to follow-up, it was planned to recruit 570 women.

A review of the literature on the topic of nipple damage found a lack of consistency in assessment of nipple damage [18]. Many reports have not provided a clear description of the assessment process. Some of the more recent studies have provided a more detailed description, such as Brent et al's Nipple Attribute Score and Duffy et al's Nipple Trauma Index [19,20]. The Nipple Trauma Index used by Duffy and colleagues in Western Australia appeared to be useful, however a request for more information about this instrument was not successful (E. Duffy, email 28 February 2001) [20].

Our definitions of nipple damage are as follows: mild 1 or 2 mm wide; moderate 3–9 mm wide; severe: greater than 10 mm wide and / or yellow colour visible in crack. In addition to a clinical assessment, a more permanent record of nipple damage was created using digital photography. It was planned for the photographs to be reviewed independently by three lactation consultants, in order to allow a thorough assessment of nipple damage and changes over time, rather than relying on the clinical assessment alone. (As the trial ended prematurely, this did not take place).

Furthermore, although the WHO defines mastitis as an inflammation of the breast [21], there is no generally agreed definition of mastitis for research purposes. The definition of mastitis used for this study was that a woman reported:

• at least two breast symptoms (pain, redness, lump) and

• at least one of fever or 'flu-like symptoms.

### Foreseen problems

#### Multi-centred trial

As we intended to recruit over 500 women we planned a multi-centred trial, involving a number of public and private maternity hospitals in inner Melbourne. All hospitals provide a breastfeeding clinic staffed with International Board Certified Lactation Consultants for women having breastfeeding difficulties following hospital discharge. The public hospitals, where women tend to have shorter hospital stays, also provide home visits by domiciliary midwives post discharge. It was foreseen that there would be replication in the requirements of the hospital ethics committees and logistical difficulties for one researcher (LA) to conduct the study on multiple sites.

Each hospital had its own research ethics committee (or committees) and different forms to submit (at the time of this study). Approval was obtained from the Ethics Committees at La Trobe University (20/11/2000), Royal Women's Hospital (6/9/2000), Mercy Hospital for Women (12/2/2001), Frances Perry House (23/8/2001), Freemasons Maternity Hospital (15/3/2001) and Cabrini Private Hospital (24/04/02). One private hospital did not appear to have a procedure in place to deal with a research proposal. Negotiations continued with this hospital from late 2000 until mid-2002 when the hospital insisted that we sign a Sponsor Indemnity Form, which the university advised us against.

The researcher visited the postnatal wards and breastfeeding clinics of these hospitals each day or second day and asked a senior member of the nursing staff if there were any breastfeeding women with damaged nipples in the ward. The staff member introduced the researcher to the woman in order to inform the woman about the study and invite her to participate in the trial. Also, the researcher asked the domiciliary midwives to inform women at home with a cracked nipple about the trial. If the woman were interested in the study, the midwife gave the researcher the woman's name and phone number. After a telephone discussion, the researcher would visit her at home to assess her eligibility.

Thus, the researcher was visiting a number of hospitals on a daily basis and making home visits to potential participants and follow-up visits to participants one week after recruitment. Therefore, if the researcher was going to be unavailable one week, she could not recruit women the week prior (as she would not be able to follow them up).

#### Funding

All potential participants had a specimen collected from their nipple crack for culture and sensitivity. As this was collected for the purpose of research rather than clinical practice, it was necessary to seek funding for the cost of the microbiological assessment. We intended to recruit 570 women, therefore substantial funds were required. A number of applications (seven) were submitted to local, national and international funding bodies in 2001. A funding application to the Medical Research Foundation for Women and Babies for 2002 was successful (A$15,000).

#### Delay between recruitment and randomization

We recognized that there would be a delay between recruitment (when the initial data and nipple specimen were collected) and randomization (when the result was available). The Microbiology laboratory faxed the result to the researcher (or the researcher contacted the laboratory on weekends). However, the minimum time was 2 days for the laboratory to identify *S. aureus *and up to 6 days in one instance (mean 3.6).

The delay meant that women would be at home when the results were available and the researcher was required to visit the participant at her home to deliver the capsules. In addition to the inconvenience, a small number of women had already developed mastitis by the time the researcher contacted her with the result.

### Unforeseen problems

#### Production of placebo capsules

It was expected that a local company specializing in the preparation of placebos for drug trials would prepare the identical capsules. A common practice is to cover the active capsule with a larger capsule; participants are unaware if their capsule contains the active capsule or an inert substance. However, when the company realized that the active capsule contained a penicillin-like drug they were unable to participate, as they do not have a license for penicillin. Finally, the pharmaceutical company, CSL Ltd, provided us with identical empty capsules as well as active flucloxacillin capsules. A pharmacy technician at the pharmacy department at the Royal Women's Hospital opened each capsule manually and inserted glucose powder. Randomisation was conducted in blocks of ten, stratified according to hospital. Ten bottles were prepared for each hospital prior to the trial commencing (further capsules were not needed).

#### Participation

**Figure 1:**
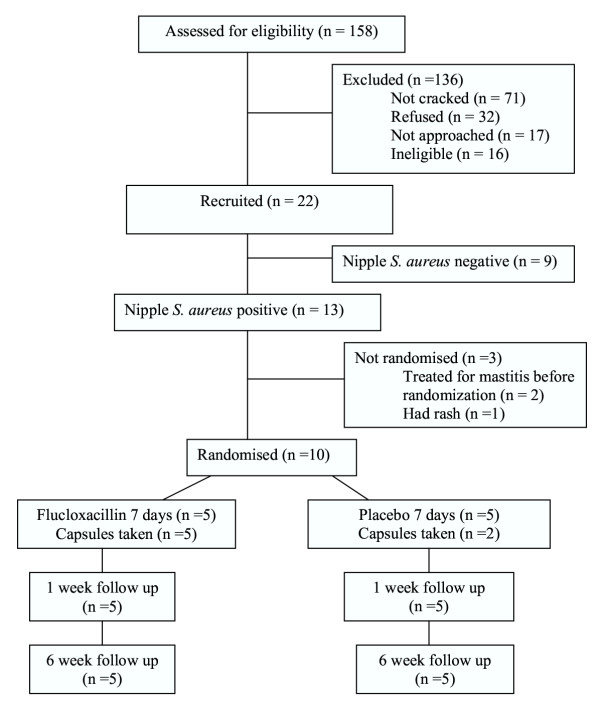
ROBIn Trial Profile

Not all the women who were eligible for the trial were interested in taking part (see Figure 1, ROBIn Trial Profile). Some women expressed a reluctance to take antibiotics, others were overwhelmed with the difficulties they were experiencing and preferred not to participate in a trial. The researchers had previously conducted studies involving breastfeeding women which had high rates of participation and had expected women to be more interested in taking part in a trial that aimed to prevent mastitis. We should have expected a lower participation rate as this study involved the possibility of taking a medication, in particular an antibiotic.

#### Less than anticipated incidence of cracked nipples

A total of approximately 17,000 women give birth in these hospitals each year. We estimated that 80% of women start breastfeeding, 5% develop cracked nipple(s), 80% would be eligible and 95% would agree to participate, thus there would be 537 eligible women per year. We anticipated that we would recruit approximately ten women with cracked nipples per week. It would therefore take 57 weeks (57 × 10) to recruit the total sample.

However, recruitment was slow, as very few women were identified with damaged nipples. Hospital staff made unsolicited remarks that nipple damage was seen much less frequently than in the past. Midwives have been trained to help women position the baby and attach the baby at the breast; women are reporting the presence of nipple pain and any nipple damage is usually identified at an early stage. In the past, women may have continued to breastfeed with poor attachment of the baby to the breast, resulting in more severe damage, whereas at the time of the study maternity staff were likely to suggest "resting" the damaged nipple and expressing the milk by hand or electric pump until the damage had healed.

## Results

Recruitment began at two hospitals in November 2001, two others in February 2002 and a fifth hospital in June 2002. Recruitment was slow as few women had damaged nipples. During the months of the trial, the rate of recruitment decreased rather than increased. Additionally, the flucloxacillin supplied by CSL were labeled to use before the end of November 2002. Therefore it was decided to stop recruiting, once a twelve-month recruiting period had elapsed. The trial stopped recruiting on the 14^th ^November 2002.

Of the 158 women referred to the study as possible participants, 48 women were eligible (ie they had a cracked nipple, were not allergic to penicillin, did not have concurrent "nipple thrush" and had adequate English). Twenty-six of these women refused (10 not interested, 9 didn't want to take antibiotics, 7 other reason given). Therefore, 22 were potentially eligible in that they had at least one cracked nipple and consented to take part in the trial if the results of the nipple swab confirmed *S. aureus*. Thirteen of the nipple cultures were positive and ten women were randomized to receive flucloxacillin (n = 5) or placebo capsules (n = 5). Two women had already developed mastitis prior to receiving the results and the third woman had developed a rash and did not want to take the capsules. All women were followed-up at one week and six weeks. Of the ten women in the RCT, one woman in the placebo group developed mastitis (not in the first week of the trial, baby was 32 days old, 28 days after randomization). Three women reported that they had not taken the capsules. When the study was unblinded it showed that all three were in the placebo group.

## Discussion

This trial experienced a number of problems, both foreseen and unforeseen. In the trial conducted by Livingstone and Stringer, there is no mention of women refusing to participate in the study or not taking the treatment they were allocated [13]. It is not reported if any woman developed mastitis in the period between collection of the swab, the clinician receiving the result and the woman being given her allocated treatment regime – indeed the paper does not state that women had to return to the breastfeeding clinic for this. Possibly, women attending a breastfeeding clinic are more likely to comply with treatment regimes than women who are invited to participate in a trial.

We thought the estimate of 5% of breastfeeding women developing a cracked nipple was a conservative estimate. For example, in Western Australia, Duffy et al had found that 6% of women in their intervention group had cracked nipples, compared to 69% in their control group [20]. However, on visiting the postnatal wards and breastfeeding clinics in inner Melbourne, it was not unusual to find that the staff were unable to identify any women with damaged nipples. And of the women who were assessed, more than half did not have a cracked nipple. Therefore, nipple damage appears to be uncommon in breastfeeding women in Melbourne.

## Conclusions

In retrospect, we should have conducted a pilot or feasibility study before commencing the trial. The appropriate use of antibiotics for breastfeeding women with cracked nipples still needs to be tested. We hope our experience will be useful for others planning trials of mastitis or nipple damage.

## Competing interests

None declared.

## Authors' contributions

All authors contributed to the design of the trial, LA reviewed the literature, conducted the trial, and wrote the first draft of the paper. All authors approved the final draft of the paper.

## Pre-publication history

The pre-publication history for this paper can be accessed here:


